# Identification of the *FGB* gene polymorphism and analysis of its association with fat deposition traits in Hu sheep

**DOI:** 10.1080/10495398.2024.2344207

**Published:** 2024-04-26

**Authors:** Lijuan He, Weimin Wang, Xiaojuan Wang, Deyin Zhang, Yukun Zhang, Yuan Zhao, Liming Zhao, Xiaolong Li, Jiangbo Cheng, Dan Xu, Zongwu Ma, Xiaobin Yang, Zhiqiang Huang, Youxin Cai, Xiaoqiang Liu, Zhanyu Chen, Xiuxiu Weng, Changchun Lin, Ping Gong, Xiaoxue Zhang

**Affiliations:** a College of Animal Science and Technology, Gansu Agricultural University, Lanzhou, Gansu, China; b State Key Laboratory of Herbage Improvement and Grassland Agro-ecosystems, College of Pastoral Agriculture Science and Technology, Lanzhou University, Lanzhou, Gansu, China; c Institute of Animal Husbandry Quality Standards, Xinjiang Academy of Animal Science, Urumqi, China

**Keywords:** Association analysis, Hu sheep, single nucleotide polymorphism, fat deposition, FGB gene

## Abstract

As a crucial economic trait, fat deposition is directly related to carcass quality and feed efficiency in sheep. The purpose of this study was to investigate the polymorphisms of the *FGB* gene related to fat deposition and detect the expression features of the *FGB* gene in different adipose tissues of sheep by using Sanger sequencing, MassARRAY® SNP technique, and quantitative real-time PCR (qRT-PCR). Results showed that in the intron region of the *FGB* gene, a SNP g. 3378953 A > T has been identified, and significant association was found between perirenal fat weight, perirenal fat relative weight, mesenteric fat weight, and mesenteric fat relative weight (*P* < 0.05). Moreover, qRT-PCR analysis showed that *FGB* was expressed in all three adipose tissues, and *FGB* gene expression level in the AA genotype was significantly lower than that in the AT or TT genotypes (*P* < 0.05). Therefore, the *FGB* gene can be used as a candidate gene to reduce fat deposition in Hu sheep breeding, and the selection of the AA genotype in Hu sheep in production practice is more conducive to improving production efficiency.

## Introduction

Hu sheep originated in Taihu Lake basin, is a unique and valuable sheep breed in China. It is widely distributed in China and is known for its rapid growth, good meat quality, strong stress resistance and other remarkable characteristics.^1–3^ Fat plays an important role in the metabolic balance of livestock.^4^ Fat deposits occur in specific locations within the body, including the abdominal cavity, intermuscular areas, subcutaneous, intramuscular, and even in the tail. The internal fat was deposited in a certain order, including perivisceral fat, intermuscular fat, subcutaneous fat and intramuscular fat.^5–8^ Fat, as one of the high-calorie foods, can maintain the basic energy required by the body when food is scarce and plays a vital role in survival.^9^ In recent years, with the upgrading of consumption and changes in lifestyle, the public's demand for meat products has shifted from the pursuit of quantity to quality, safety and nutrition.^10^ Mutton is widely loved by consumers because of its unique taste and flavor, balanced nutritional value and geographical culture.^11^ Adipose tissue is essential for the body's energy metabolism, storing excess calories and maintaining proper energy balance and overall health. When fat is deposited excessively, it will lead to obesity, metabolic disorders and inflammatory reactions, causing some negative effects on the body. The accumulation of fat during the feeding process not only increases the cost of feeding but also serves as an important indicator for evaluating the production performance of sheep. Intramuscular fat content is closely related to the quality of mutton and affects the physicochemical and sensory properties of mutton.^12^ But too much of the other fat (visceral fat, tail fat) can directly affect the growth rate and ultimately affect the economic benefits of sheep farms.^13^ Therefore, the reduction of fat deposition has emerged as crucial research of study within the field of sheep breeding.

Marker-assisted selection (MAS) improves breeding selection by using phenotypic, genealogical, and genetic markers for more efficient and accurate selection. Single nucleotide polymorphisms (SNPs) are important genetic markers due to their connection with economically significant traits in animal husbandry. In sheep, studies have showed associations between SNPs and genes related to fat deposition. For instance, *RAP1GAP* and *rBAT* polymorphisms can be used as candidate molecular markers to reduce fat deposition in sheep tail.^14^ Similarly, *TRAPPC9*g.57654 A > G and *BAIAP2*g.46061 C > T were significantly correlated with tail fat.^15^ In addition, *HMGA1*g.5312 C > T was significantly correlated with tail fat.^16^

Fibrinogen beta chain (FGB) is a glycoprotein that is synthesized and secreted by liver cells. It is encoded by the *FGB* gene and plays a significant role in the development of cardiovascular diseases.^17^^,^^18^ Fibrin is a key element of blood clots and is important in the process of blood coagulation.^19^ Furthermore, fibrinogen also plays a crucial role in various physiological and pathological processes, including inflammation.^20^ In studies of human fat, it has been found that the *FGB* gene affects platelet Ca^2+^ levels, which can hinder early stages and encourage late adipocyte differentiation.^21^ The study shows the association between the *FGB* − 455 G > A genetic polymorphism and lipid metabolism in individuals with hypertension.^22^ In addition, the *FGB* gene was identified as one of the nodes with the highest score in an analytical cluster of diabetes gene networks.^23^ *FGB* is also an active endocrine factor in the gene network that regulates abdominal visceral fat. Additionally, it has been shown to play a role in promoting antimicrobial immune responses through both innate and T-cell-mediated pathways.^24^ The role of *FGB* in anti-tumor therapy has been recognized in recent years. SNP in *FGB* is significantly associated with fibrinogen levels,^25^ and the increase in fibrinogen synthesis may be mediated by mechanisms involved in insulin resistance,^26^ acting in this way on fat deposition. Both fibrinogen and fibrinogen-like-protein 2 (Fgl2) belong to the fibrinogen superfamily and may play similar biological functions. Studies have found that Fgl2 acts on lipid metabolism through specific pathways.^27^ At present, the mechanism of the gene involved in sheep fat deposition has not been reported yet, and its function remains unclear. Therefore, the purpose of this study was to explore the relationship between *FGB* gene and fat deposition in Hu sheep. We identified a mutation site of *FGB* gene by PCR amplification, Sanger sequencing technology and MassARRAY genotyping technology, and analyzed the association between this gene and fat deposition traits, and studied the expression levels of the *FGB* gene in different adipose tissues and different genotypes in adipose tissues. This study will provide a new molecular marker site for reducing fat deposition in Hu sheep.

## Materials and methods

### Moral statement

This study strictly followed the approved procedures of the Animal Care and Use Committee for Biological Research of Gansu Province, China. The sample collection and experimental program were approved by the Ethics Committee of Gansu Agricultural University (Animal Experimentation License No. 2012-2-159).

### Animals, samples collection and DNA extraction

In this study, 1071 healthy male Hu sheep were selected from Defu Agricultural Technology Co., LTD. These sheep are carefully selected because they have complete genealogical information. And vaccinations are given on time (before 56 days of age) according to standard procedures. The lambs were selected from different farms and birthplaces are cited in Lin et al.^28^ and Zeng et al.^29^ The experiment consisted of three phases: transition period (14 days), pretrial period (10 days), and final trial period (100 days). All experimental animals were kept and managed under the same conditions. In addition, all Hu sheep are weighed and slaughtered at 180 days. Abstain from food and water for 12 hours before slaughter to ensure accurate weight measurement. After slaughter, tail, mesenteric and perirenal fat were accurately extracted and weighed, and some tissues were collected and frozen in a cryostorage tube and stored in an ultra-low temperature refrigerator at −80 °C for subsequent RNA extraction. DNA was successfully extracted from the blood of 1071 experimental Hu sheep (6 months) using the method described in the Kit (TransGen Biotech) and stored in a −20 °C refrigerator.

### SNP identification and genotyping

Primer 5.0 and Oligo 7.0 software were utilized to design primers based on the ovine *FGB* gene sequence in GenBank (NC_040268.1). The designed primers are presented in Table 1. Gradient PCR was performed using a 10 *μ*L reaction system at the recommended temperature provided by the manufacturer. The PCR reaction mixture (10 *μ*L) was: 2 × Easy Taq PCR Super Mix (Transgen) 5 *μ*L, positive and reverse primers 0.32 *μ*L each, dNTPs 0.4 *μ*L, ddH_2_O 4 *μ*L. The amplification products obtained at the optimal temperature were then sequenced to determine the mutation site of the *FGB* gene. The PCR amplification using the system with the optimal temperature (system 35) was performed with the following reaction components: 17.5 *μ*l Easy Taq PCR Mix, 1.12 *μ*l each primer, 1.4 *μ*l DNA, and 14 *μ*l double-distilled water. The PCR amplification process followed a set of cycle parameters. These parameters included a predenaturation step at 94 °C for 5 minutes, followed by denaturation at 94 °C for 30 seconds. Subsequently, annealing occurred at 51 °C for 30 seconds, followed by extension at 72 °C for 30 seconds. This cycle was repeated 35 times in total. Finally, a final extension step occurred at 72 °C for 5 minutes. The SNP identified within the *FGB* gene was genotyped using MassARRAY.^30^ The genotyping primers used can be found in Table 2.

**Table 1.** The sequence of the sheep *FGB* gene primer and PCR conditions.

Gene	primer	Primer sequence (5'–3')	Product length	Optimum temperature
*FGB*	*FGB*-F	CACGTTACCATCACTGTCA	470 bp	51 °C
	*FGB*-R	TCTTCTACACTCTTTCGGACT		
*FGB*	*FGB*-mF	ATGTTCTTCAGCACGTACGAC	112 bp	58 °C
	*FGB*-mR	CTGCGTGACATCGGTTATACCA		
*GAPDH*	*GAPDH*-F	AGATGGTGAAGGTCGGAGTG	188 bp	54 °C
	*GAPDH*-F	GTTCTCTGCCTTGACTGTGC		

**Table 2.** MassARRAY genotyping primer.

Gene	Primer	Primer sequence (5'-3')
*FGB*	Primer Allele FAM	CTGTAGGACACAACACACCCTAT
	Primer Allele HEX	CTGTAGGACACAACACACCCTAA
	Primer Common	ATGTGCTTGTTTTGCAAATACCTCAA

### Tissue expression analysis of FGB

Nine experimental sheep were selected according to different genotypes for tissue expression analysis. Total RNA from each tissue was extracted using Transzol (TransGen Biotech, China). 2^-ΔΔCT^ method was used to calculate relative gene expression.^31^ Primers for qRT-PCR are shown in Table 1, using *GAPDH* as the reference gene.

### Statistical analysis

Genotypes and traits associated with fat deposition were analyzed using a general linear model (GLM) with multiple regression models, which was defined as:

$$Y_{\text{ijk}}=\mu +G_{i}+F_{j}+S_{m}+\varepsilon_{\text{ijk}}$$

In this study, Y*_ijk_* denotes the observed phenotype value for fat deposition traits, while *μ* represents the mean value. Genotype-specific effects are given by G*_i_* (i is the index for different genotypes), and F*_j_* accounts for farm effects (j = 1, 2….5). Additionally, S*_m_* represents the impact of the season (m = winter, summer) ε*_ijk_* represents the residual error for each observed trait value. A statistically significant result was determined at the *P* < 0.05 level. Use the website(https://www.hiblup.com/tutorials)to calculate the frequency of genotypes and alleles. SPSS 25.0 software and Excel were used for all statistical analyses.

## Results

### Descriptive statistics between traits

The results of the descriptive statistics for all indicators can be found in Table 3. The coefficient of variation (CV) for all traits was found to be greater than 15%. The coefficients of variation of perirenal fat weight, perirenal fat relative weight, mesenteric fat weight, mesenteric fat relative weight, tail fat weight, tail fat relative weight (body weight), tail fat relative weight (carcass), and carcass weight were 52.19%, 45.21%, 39.23%, 32.29%, 30.73%, 26.14%, 25.46%, and 15.36%, respectively. The results indicate that there were significant phenotypic differences observed in fat deposition traits within the experimental population.

**Table 3.** Descriptive statistics of fat deposition traits.

Traits	Max	Min	Mean	SD	CV (%)
The weight of perirenal fat (kg)	2.28	0.07	0.71	0.37	52.19
The relative weight of perirenal fat	0.04	0.00	0.01	0.01	45.21
The weight of mesenteric fat (kg)	3.06	0.21	1.17	0.46	39.23
The relative weight of mesenteric fat	0.06	0.01	0.02	0.01	32.29
The weight of tail fat (kg)	3.44	0.34	1.55	0.48	30.73
The relative weight of tail fat (body weight)	0.07	0.01	0.03	0.01	26.14
The relative weight of tail fat (Carcass)	0.15	0.02	0.06	0.01	25.46
Carcass weight (kg)	42.30	11.60	26.63	4.09	15.36

Note: Tail fat relative weight (body weight) = tail fat weight/live weight before slaughter × 100%.

Tail fat relative weight (carcass) = tail fat weight/Carcass weight × 100%.

### SNP identification of the FGB gene

**Figure 1.**
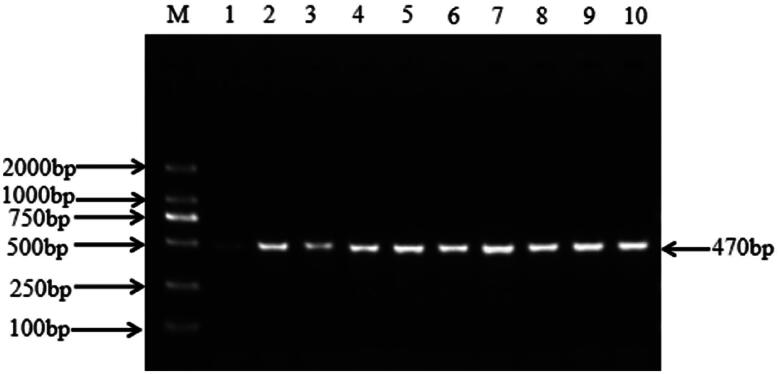
*FGB* gene PCR amplification products. *Note*: M: DL2000 DNA marker; 1-10: PCR products.

**Figure 2.**
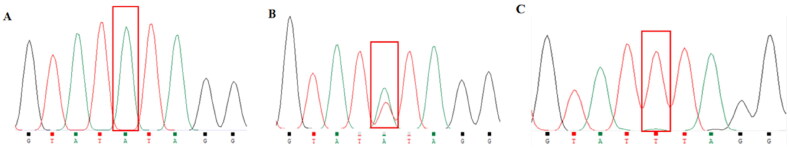
Sequencing peak of *FGB* gene in Hu sheep. (A): AA type, (B): at type, (C): TT type. Red box: the mutation site is *FGB* g.3378953 A > T.

**Figure 3.**
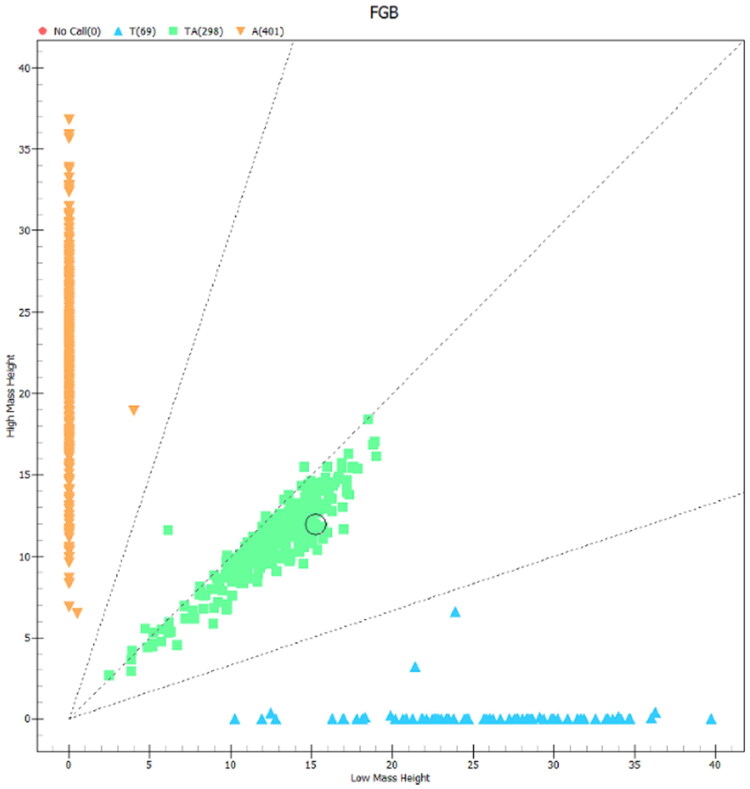
MassARRAY® SNP-based single nucleotide polymorphism (SNP) genotyping of *FGB* g.3378953 A > T in Hu sheep population.

The 470 bp fragment of the *FGB* gene was successfully amplified by the primer shown in Table 1 (shown in Figure 1). The gene polymorphism in Hu sheep was identified by sequencing the DNA pool's PCR products. The sequencing results showed the presence of the g.3378953 A > T mutation in intron 3 (Figure 2). MassARRAY analysis were used to genotype the SNP at g.3378953 A > T of *FGB*. Three genotypes (AA, AT and TT) were detected after genotyping (Figure 3).

### Genetic indexes of FGB gene

Three genotypes were identified and the corresponding genetic parameters were calculated (the genotypic frequency, allelic frequency, effective allele number (Ne), heterozygosity (He), and polymorphism information content (PIC)) are shown in Table 4.

**Table 4.** The genetic type frequency, equal gene frequency, and genetic diversity of the gene types of *FGB* SNP sites.

Loci	Genotype	Genotype frequency	Allele	Allele frequency	Ne	Ho	He	PIC	PHWE
*FGB* g.3378953 A > T	AA (547)	0.53	A	0.72	1.68	0.60	0.40	0.32	0.59
	AT (396)	0.39							
	TT (85)	0.08	T	0.28					

Note: Expected heterozygosity (He), expected homozygosity (Ho), effective allele number (Ne), polymorphism information Content (PIC), and P-value of Hardy-Weinberg equilibrium (PHWE).

For *FGB* g.3378953A > T locus, genotype frequencies for AA, AT, and TT were 0.53, 0.39 and 0.08, respectively. The polymorphism information content (PIC) of *FGB* was 0.32, indicating medium genetic diversity in Hu sheep (0.25 < PIC < 0.5). The effective allele number (Ne) was 1.68, indicating a moderate level of genetic diversity in the Hu sheep population. The expected homozygosity (Ho) was calculated to be 0.60, indicating that there is a 60% chance of an individual being homozygous at this locus. The expected heterozygosity (He) was found to be 0.40, indicating a 40% chance of an individual being heterozygous at this locus. The Hardy-Weinberg equilibrium (PHWE) value of 0.59 suggests that the observed genotype frequencies are close to the expected frequencies, indicating that the population is in genetic equilibrium at this locus.

### Association analysis between the FGB gene and fat deposition traits in Hu sheep

Association analysis of fat deposition traits in Hu sheep showed that the *FGB* g.3378953 A > T gene polymorphism appeared a noteworthy association with perirenal fat and mesenteric fat weight (*P* < 0.05). The AA genotype showed significantly lower perirenal and mesenteric fat weights compared to AT and TT genotypes (*P* < 0.05), demonstrating a clear difference (Table 5).

**Table 5.** Association analysis of the *FGB* g.3378953 a > T SNP in sheep.

Item	AA (547)	AT (396)	TT (85)	P-Value
The weight of perirenal fat (kg)	0.682 ± 0.016^b^	0.737 ± 0.019^a^	0.776 ± 0.040^a^	0.020
The relative weight of perirenal fat	0.014 ± 0.000^b^	0.015 ± 0.000^a^	0.015 ± 0.001^a^	0.017
The weight of mesenteric fat (kg)	1.135 ± 0.020^b^	1.216 ± 0.023^a^	1.234 ± 0.050^a^	0.013
The relative weight of mesenteric fat	0.023 ± 0.000^b^	0.024 ± 0.000^a^	0.024 ± 0.001^a^	0.005
The weight of tail fat (kg)	1.534 ± 0.020	1.556 ± 0.024	1.63 ± 0.051	0.466
The relative weight of tail fat	0.031 ± 0.000	0.032 ± 0.000	0.033 ± 0.001	0.202
The relative weight of tail fat (Carcass)	0.058 ± 0.001	0.058 ± 0.001	0.06 ± 0.002	0.430
Carcass weight (kg)	26.556 ± 0.174	26.663 ± 0.204	27.14 ± 0.441	0.516

Note: Values for phenotypic data are shown as mean ± standard error. Different superscript lowercase letters in the same column indicate significant differences (*P* < 0.05).

### Expression of the FGB between different genotypes

**Figure 4.**
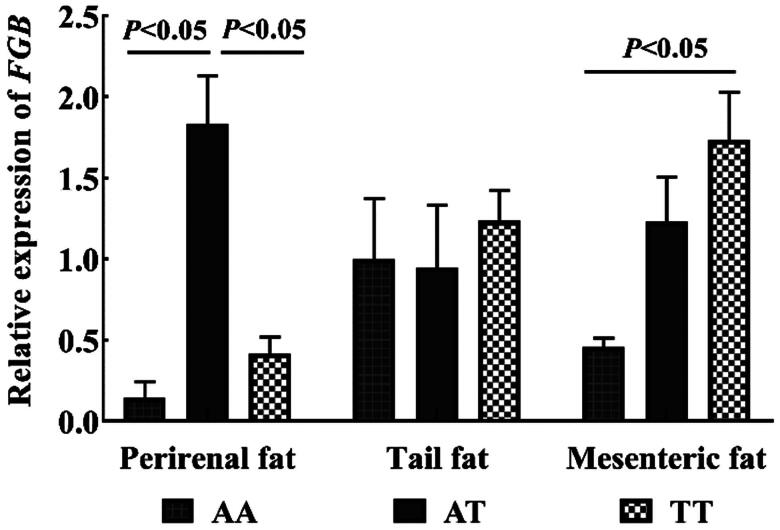
*FGB* mRNA expression profile in Hu sheep fat tissues.

The mRNA levels of *FGB* in perirenal fat, tail fat, and mesenteric fat were detected by qRT-PCR. The findings presented in Figure 4 demonstrate that the perirenal fat expression of the AA genotype was significantly decreased compared with that of the AT genotype (*P* < 0.05). Additionally, the mesenteric fat of the AA genotype was remarkably less than TT genotype (*P* < 0.05), while no significant difference was observed among the three genotypes in tail fat.

## Discussion

In recent years, there has been a raising awareness of various diseases, and diet plays a crucial role in maintaining health. As a sough-after meat choice, mutton has gained significant attention from consumers who prefer low-fat options. In the modern mutton industry system, high fat deposits not only require increased feed consumption but also have a negative impact on the quality and taste of mutton. In this study, the coefficient of variation of all fat deposits-related traits was higher than 20%, showing strong selection potential, which is consistent with the results of previous studies.^15^ Fibrinogen is an important plasma protein secreted by the body and consists of three (Aα, Bβ, γ) polypeptide chains.^32^ The fibrinogen β chain (FGB) is the rate-limiting portion of mature fibrinogen production.^33^ Previous studies have found that levels of fibrinogen may be associated with obesity (body mass index).^34^^,^^35^ An intron mutation site *(FGB* g.3378953 A > T) was identified by Sanger sequencing. Although it does not change amino acids, intron mutations have been shown to affect gene function by altering mRNA structure or function,^36^^,^^37^ post-transcriptional splicing, protein conformation, and translation efficiency.^38^^,^^39^ Therefore, we used low-cost typing techniques to classify them in the expanded test population, and the results showed that there were polymorphism and unbiased in this test population, so it was used for subsequent association analysis. Finally, association analysis showed that *FGB* polymorphism site was significantly correlated with fat deposition traits (perirenal fat, mesenteric fat). In one study, it was observed that the majority of patients who did not utilize lipid-lowering medications exhibited higher levels of fibrinogen in comparison to those who did.^40^ The expression level of *FGB* gene in 3 tissues of the study was also consistent with that of the present study, and there was a positive correlation between fat deposition and fibrinogen expression level. Single nucleotide polymorphism (SNP) is an important source of genomic variation. The large amount of genomic data and high-throughput typing platform promote single nucleotide polymorphisms (SNP) as the best DNA markers for genome selection studies, which not only improves breeding efficiency, but also provides simple and reliable methods.^41^

Reducing fat deposits enables sheep to allocate more energy to growth, improving economic traits such as feed efficiency and carcass quality. The tissue expression results showed that the *FGB* gene was expressed in three adipose tissues. This finding is consistent with a previous study conducted by Kathrin Halli et al.,^42^ who also identified the *FGB* gene as a potential candidate gene for maternal influence on intramuscular fat. The results showcase that the *FGB* gene likely perform an increasingly important role in fat deposition regulation. Excessive deposition of fat in visceral tissue has been associated with elevated blood pressure, increased risk of thrombosis, and heightened inflammatory status in the body.^43^ Fibrinogen demonstrates a significant positive associated with a multitude of obesity markers, encompassing blood pressure, visceral and subcutaneous fat. As a result, it can be concluded that visceral adipose tissue is a more powerful predictor of cardiovascular risk.^44^ Visceral fat is more likely to cause an inflammatory response. Liu et al.^45^ found that the accumulation of fat around the colon is linked to a higher risk of developing colon polyps. Studies have shown that adipose tissue is the coordinator of immune and metabolic processes.^46^ Smoking, high blood pressure and obesity are typical factors for thrombosis.^47^ There is strong evidence that platelets and vascular endothelial cells play a central role in the pathogenesis and progression of hypertension.^48^ Endoplasmic reticulum (ER) stress exerts notable effects on adipose tissue (visceral adipose tissue), liver, and skeletal muscle in individuals who have obesity.^49^ Furthermore, obesity is linked to immune system impairment and is a significant contributing element to various forms of cancer.^50^ The *FGB* gene is not only a candidate gene for intramuscular fat but also an active secretory factor in visceral fat. Additionally, it plays a role in immune and inflammatory responses. Visceral fat (perirenal fat, mesenteric fat) has also been shown to be associated with immune and inflammatory responses. Therefore, the *FGB* gene may regulate the body's immune and inflammatory response through visceral fat.

In the previous part, we analyzed the genotypes and fat deposition traits of 1072 Hu sheep. These results indicated that the *FGB* g.3378953 A > T polymorphism was significantly associated with perirenal fat weight, perirenal fat relative weight, mesenteric fat weight, and mesenteric fat relative weight. This is consistent with the report of Bo M et al.,^51^ which shows that fibrinogen level is closely related to body fat content and metabolic variables. Therefore, the polymorphic loci of *FGB* gene may serve as promising molecular biomarkers and have important research value for Hu sheep breeding. To improve the overall quality and productivity of the Hu sheep population, benefiting both farmers and consumers. However, the specific pathways and mechanisms by which the *FGB* gene affects fat deposition in Hu sheep need to be further verified by relevant experiments.

## Conclusion

In this study, the measurement and statistics of perirenal fat weight, mesenteric fat weight and tail fat weight found that the coefficient of variation was more than 20%, so it has great potential for selection. The *FGB* gene mutation site (*FGB* g.3378953 A > T) of Hu sheep was identified by sanger sequencing. MassARRAY genotyping identified three genotypes at this mutation site. Association analysis of fat deposition traits showed that the perirenal fat weight and mesenteric fat weight of AA type individuals were significantly lower than those of AT type individuals and TT type individuals, which was dominant alleles. Tissue expression analysis showed that *FGB* gene was widely expressed in adipose tissue, and the expression level was lowest in visceral adipose tissue of AA genotype individuals. Therefore, this mutation site has the potential to be used as a molecular genetic marker to reduce the fat deposition trait of Hu sheep through targeted breeding efforts.
